# An in silico toolbox for the prediction of the potential pathogenic effects of missense mutations in the dimeric region of *h*RPE65

**DOI:** 10.1080/14756366.2022.2162047

**Published:** 2023-01-11

**Authors:** Giulio Poli, Gian Carlo Demontis, Andrea Sodi, Alessandro Saba, Stanislao Rizzo, Marco Macchia, Tiziano Tuccinardi

**Affiliations:** a Department of Pharmacy, University of Pisa, Pisa, Italy; b Department of Neurosciences, Psychology, Drug Research and Child Health Eye Clinic, University of Florence, AOU Careggi, Florence, Italy; c Department of Surgical Pathology, Molecular Medicine and of the Critical Area, University of Pisa, Pisa, Italy; d Ophthalmology Unit, Fondazione Policlinico Universitario A. Gemelli IRCCS, Rome, Italy; e Catholic University Sacro Cuore, Rome, Italy; f Consiglio Nazionale delle Ricerche, Istituto di Neuroscienze, Pisa, Italy

**Keywords:** RPE65, variant of uncertain significance (VUS), molecular dynamics, missense mutations

## Abstract

*h*RPE65 is a fundamental enzyme of the retinoid visual cycle, and many missense mutations affecting its expression or function are associated with a wide range of diseases. Many *h*RPE65 missense mutations lack a clear pathogenicity classification or are labelled as VUS. In this context, we recently developed a protocol based on µs-long molecular dynamics simulations to study the potential pathogenic effect of *h*RPE65 missense mutations. In the present work, the structure-based protocol was integrated with a *h*RPE65-tailored consensus bioinformatics strategy, named ConPath, that showed high performance in predicting known pathogenic/benign *h*RPE65 missense mutations. The combined strategy was used to perform a multi-level evaluation of the potential pathogenicity of 13 different *h*RPE65 VUS, which were classified based on their likelihood of pathogenic effect. The obtained results provide information that may support the reclassification of these VUS and help clinicians evaluate the eligibility for gene therapy of patients diagnosed with such variants.

## Introduction

Opsins are a group of G protein-coupled receptors capable of capturing photons and thus initiate visual perception in rod and cone photoreceptors of the retina, using 11-cis-retinaldehyde (11-cis-RAL) as a chromophore^1^. Absorption of a photon by a rod- or cone-opsin pigment induces isomerisation of 11-cis-RAL to all-trans-retinaldehyde (all-trans-RAL) that dissociates from the bleached pigment, rendering it insensitive to light. To restore light-sensitivity, the all-trans-RAL is subjected to a multistep process termed retinoid visual cycle that reisomerize it to 11-cis-RAL, which recombines with apo-opsin to form a new pigment molecule.^2^ The first catalytic step of the visual cycle is the reduction in photoreceptors of all-trans-RAL to all-trans-retinol (all-trans-ROL) that is released from the photoreceptors and is esterificated by lecithin:retinol acyltransferase (LRAT) to all-trans-retinyl esters (all-trans-RE) in the retinal pigment epithelium (RPE). Then, the retinal pigment epithelium 65 kDa protein (RPE65) catalyses the trans-cis isomerisation reaction, taking all-trans-RE as substrates and converting them, through hydrolysis and alkene isomerisation, into 11-cis-retinol (11-cis-ROL) that is finally oxidised to 11-cis-RAL by 11-cis-retinol dehydrogenase enzymes and then shuttled back to photoreceptors to regenerate ground-state visual pigments^3^.

The human RPE65 (*h*RPE65) belongs to a superfamily of enzymes known as carotenoid cleavage dioxygenases (CCDs), is abundantly expressed in RPE cells and shuttles between the cytoplasm and the endoplasmic reticulum. Interestingly, the lack of *h*RPE65 as in Rpe65^−/−^ mice is associated not just with the inability to form 11-cis-RAL, but also with a large accumulation of retinyl esters in the RPE with a slow and progressive loss of photoreceptors^4^. Regarding the RPE65 gene, numerous pathogenic variants have been identified in humans. Many of these variants introduce a nonsense or a missense mutation capable of affecting protein expression or enzyme function and are associated with a spectrum of inherited retinal diseases ranging from Leber’s congenital amaurosis (LCA) to retinitis pigmentosa (RP)^5^. Voretigene neparvovec (Luxturna™) is the first gene therapy approved for the treatment of retinal degeneration^6^; it is delivered as an adeno-associated virus (AAV2)-based genetic treatment in a subretinal injection to patients with an inherited retinal dystrophy caused by biallelic mutations in the RPE65 gene^7^. Confirmation of pathogenic *h*RPE65 mutations is essential for identifying patients for whom Luxturna™ may be advantageous. The inherent risks of a surgical procedure and anaesthesia, especially in the paediatric population, elevate the burden of proof for confirming the pathogenicity of any identified mutations. The two mutations not only should segregate to each of the parents but should also have a sufficiently high suspicion for impairing protein function. When compound heterozygous or homozygous *h*RPE65 variants of uncertain significance (VUS) are encountered, clinicians should confirm the protein dysfunction *in vitro* before proceeding with a gene therapy surgical procedure^8^^,^^9^. Up to now, more than 150 *h*RPE65 missense mutations lacking a clear pathogenicity classification or classified as VUS have been reported in public databases^10–14^. Recently, we developed an innovative *in silico* protocol based on µs-long molecular dynamics (MD) simulation studies aimed to address the issue of patients diagnosed with an *h*RPE65 VUS^15.^ The protocol was specifically calibrated and optimised for assessing the impact of missense mutations located within the dimerisation region of *h*RPE65, essential for subcellular localisation and therefore protein stability, expression and enzymatic activity^15^. In the present work, the protocol was applied to perform a thorough structure-based evaluation of the potential pathogenicity of a set of 13 different *h*RPE65 VUS associated to residues located in such protein region. Moreover, a novel, orthogonal *in silico* strategy for evaluating the potential pathogenic effect of *h*RPE65 missense VUS was developed by combining the prediction of 19 different bioinformatics tools, which showed high predictive reliability when tested using known *h*RPE65 missense mutation with known effect. This novel *h*RPE65-tailored consensus bioinformatics strategy was combined with the results of the structure-based analysis for providing a comprehensive assessment of the potential pathogenicity of the different *h*RPE65 VUS herein studied, which were classified based on the likelihood of pathogenic effect.

## Materials and methods

### Protein structure modelling

The monomeric wilt-type (WT) DMS-focussed *h*RPE65 model developed in our previous study^15^, obtained from the X-ray structure of bovine RPE65 (PDB code 3FSN)^16^, was used in this work. The monomeric models of the 13 *h*RPE65 variants herein analysed (K303N, N373S, D375N, D375H, E399K, T390I, V407A, T400S and W402S) were obtained by mutating the proper residues of the WT DMS-focussed *h*RPE65 monomeric model and automatically optimising the side chains of the mutated residues using Modeller software^17^. The 13 DMS-focussed dimeric models, corresponding to K303N-WT, N373S-WT, D375N-WT, D375H-WT, E399K-WT, T390I-WT, V407A-WT, T400S-WT and W402S-WT dimers, were obtained by combining the previously created monomeric systems based on the reference X-ray structure of bovine RPE65 (PDB code 3FSN); i.e., superimposing the proper couples of monomeric structures to the crystallographic RPE65 dimer.

### Molecular dynamics simulations

All simulations were performed using AMBER, version 20^18^, using the ff14SB force field. The solute was placed in a rectangular parallelepiped water box, by using TIP3P explicit solvent model and solvated with an 8.0 Å water cap. Sodium ions were added as counterions to neutralise the systems. Before molecular dynamics (MD) simulations, the whole systems were energy minimised using a two-stage protocol. In the first stage, 5000 steps of steepest descent (SD) followed by conjugate gradient (CG) algorithms were performed for the exclusive minimisation of the solvent, since a harmonic potential of 100 kcal/(mol·Å^2^) was applied to all solute atoms. In the second stage, 5000 additional steps of SD/CG were used to minimise the whole system, until a convergence of 0.05 kcal/(mol·Å^2^), imposing a harmonic constraint of 10 kcal/(mol·Å^2^) only on the protein α carbons. The minimised systems were used as starting conformations for the MD simulations, which were performed using Particle Mesh Ewald (PME) electrostatics, periodic boundary conditions and a cut-off of 10 Å for the non-bonded interactions. SHAKE algorithm was employed to keep all bonds involving hydrogen atoms rigid. A constant volume periodic boundary MD was carried out for 0.5 ns, during which the temperature of the systems was raised from 0 to 300 K. The systems were then pre-equilibrated through 3 ns of constant pressure simulation, using the Langevin thermostat, in order to maintain the temperature at the constant value of 300 K. During these first two MD stages, all the protein α carbons were restrained with a harmonic potential of 10 kcal/(mol·Å^2^) and the simulation was performed using a time step of 2.0 fs. An additional constant pressure MD stage of 10 ns was then performed for equilibrating the system using the hydrogen mass repartition (HMR) scheme^19^ and a time step of 4.0 fs. All the protein α carbons were restrained with the same harmonic potential of 10 kcal/(mol·Å^2^). Finally, a production stage corresponding to 3 µs of constant pressure MD simulation was performed using the HMR scheme and a time step of 4.0 fs. During the production stage, the α carbons of only 56 out of the 141 protein residues belonging to each monomeric portion of *h*RPE65 simulated in the system were subjected to the harmonic restraint of 10 kcal/(mol·Å^2^); this way, the DMS domains of the dimers and the surrounding protein residues of the adjacent β-sheets were kept totally free to move during the MD and only the external residues were restrained. The MD trajectories corresponding to the production stage of all systems were analysed using the cpptraj program^20^ implemented in Amber 20.

### Performance assessment of predictive bioinformatics tools

A wide ensemble of different algorithms for the prediction of the potential pathogenic effect of protein missense mutations, freely accessible as web tools and servers, was selected for this analysis. The pathogenicity predictions related to 26 algorithms, including BayesDel_addAF, BayesDel_noAF, ClinPred, DANN, DEOGEN2, LRT, MetaLR, MetaRNN, MetaSVM, MutationTaster, PrimateAI, REVEL, VEST4, fathmm-MKL, fathmm-XF, CADD, CADD_hg19, SIFT, Eigen-PC, Eigen, FATHMM, LIST-S2, MVP, MutationAssessor, PROVEAN and Integrated FitCons were obtained through the database of functional predictions dbNSFPv4^21^, which was accessed through the Ensembl Variant Effect Predictor web interface^22^. The predictions related to other 6 different algorithms, namely PredictSNP, PhD-SNP, PolyPhen-1, PolyPhen-2, MAPP and SNAP were accessed and obtained through PredictSNP web server^23^. The predictions related to all other algorithms, namely PANTHER, SNAP2, Meta-SNP, PON-P2, PMut, SAAFEC-SEQ, PhD-SNPg, INPS-sequence, SuSPect, MutPred2, SNPs3D, Mupro, NEI commons Mutation Search and PROST, were obtained from the corresponding freely accessible web-based applications. A dataset of 30 known *h*RPE65 missense variants reported in the ClinVar database, was used as a test set for evaluating the performance of the different bioinformatics tools in discriminating between pathogenic and benign *h*RPE65 missense mutations. Unfortunately, only three missense mutations classified as “*Benign*” are currently reported on the ClinVar database and could be used for statistical evaluations. Therefore, the dataset included 27 variants classified as “*Pathogenic*” and 3 variants classified as “*Benign*” within ClinVar. Most of the tested algorithms already provided either a binary classification of the analysed mutations or a default binary interpretation of the prediction results, which were thus used as provided (formally standardised in “Pathogenic” and “Neutral”) for statistical evaluations. The results of the tested algorithms that provided only prediction scores were converted into binary classifications using different cut-off values. In particular, a cut-off of 0 was used for the raw coding scores of Eigen and Eigen-PC, as well as for SNPs3D, INPS-sequence and PROST; a value of 0.4 was used for Integrated FitCons; 0.5 was used for REVEL, VEST4, DANN, MutPred2 and PON-P2; 0.75 for MVP; 20 for CADD and CADD_hg19 and 50 for SusPect. The performance of the different predictive methods was evaluated based on the prediction generated for the 30 known *h*RPE65, using four different statistical parameters, i.e., Precision, Specificity, Recall and Accuracy, which are defined as follows^24–26^:

$$\text{Precision}=\frac{TP}{\left(TP+FP\right)}$$

$$\text{Specificity}=\frac{TN}{\left(TN+FP\right)}$$

$$\text{Recall}=\frac{TP}{\left(TP+FN\right)}$$

$$\text{Accuracy}=\frac{TP+TN}{\left(TP+TN+FP+FN\right)}$$

TP (true positives) and TN (true negatives) correspond to the number of pathogenic and neutral mutations, respectively, correctly predicted as such; FP (false positives) represents the number of neutral variants predicted as pathogenic and FN (false negatives) represents the number of pathogenic variants predicted as neutral. Therefore, Precision indicates the percentage of correct positive predictions, while Specificity and Recall measure the ability of models in correctly predicting negative and positive instances, respectively. Accuracy takes into account all values derived from the binary classification and represents the fraction of correct predictions over all predictions. For instance, an Accuracy of 1 indicates a perfect classification (with no FP and FN), whereas a null Accuracy indicates a complete disagreement between predicted and actual classes (with no TP and TN).

### Consensus pathogenicity evaluations

The 19 different tools that showed Recall and Specificity values above or equal to 0.78 and 0.67, respectively, were selected and employed for generating the consensus-based pathogenicity prediction score named ConPath score, which combined all the different predictions obtained by the selected tools. The ConPath score corresponds to the sum of positive pathogenic predictions obtained with the different 19 methods. A ConPath score ≤9 corresponds to a prediction of a potentially neutral effect, whereas a ConPath score ≥10 corresponds to a prediction of a potentially pathogenic effect. The closer the ConPath score to 19 (the maximum value), the higher the probability of pathogenic effect; the closer the ConPath score to 0 (the minimum value), the higher the probability of neutral effect.

## Results and discussion

### Structure-based analysis of hRPE65 missense VUS

**Figure 1.**
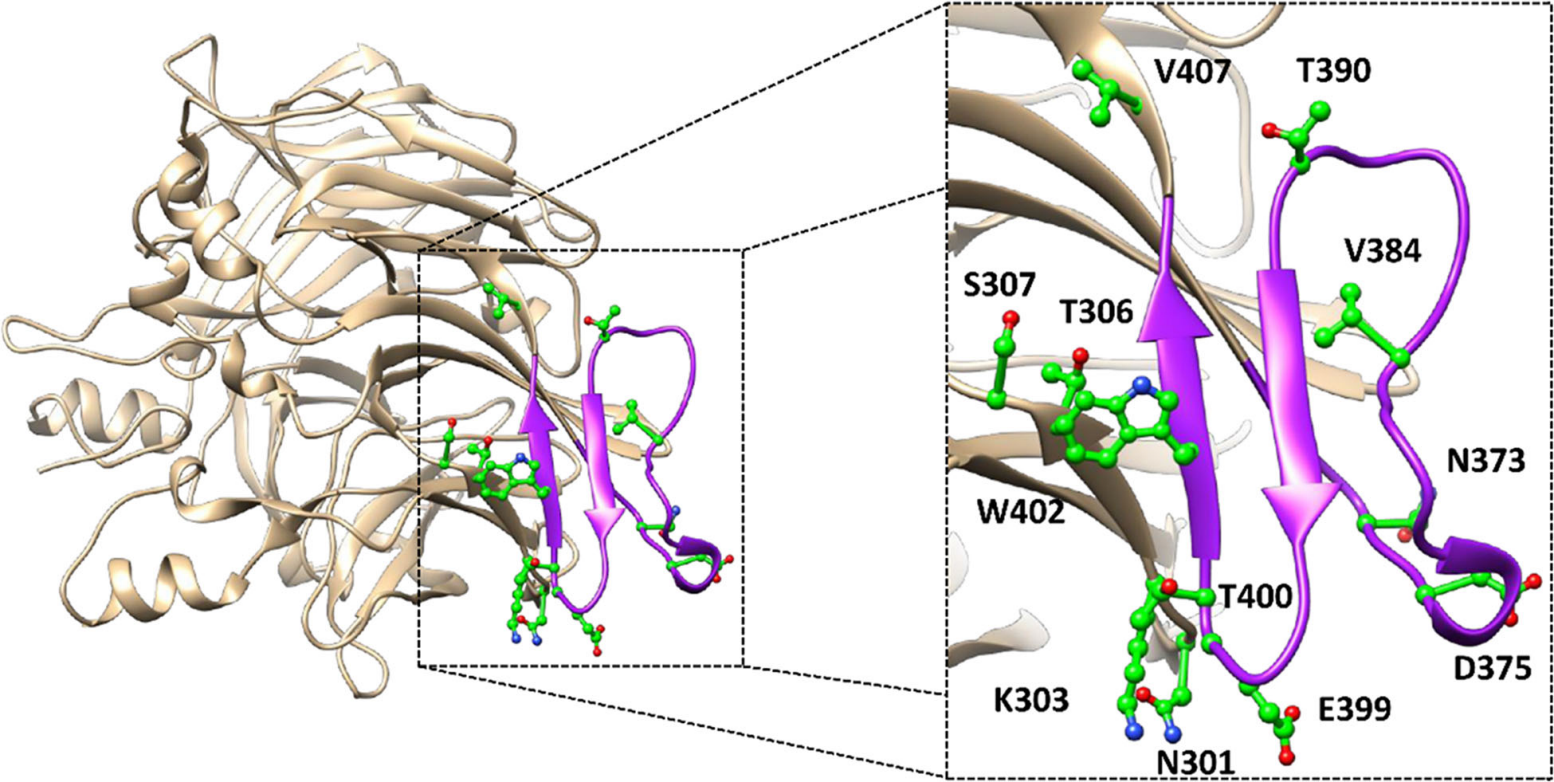
Localisation of the 12 residues (shown in green) associated to the 13 ClinVar VUS analysed. The protein sequence corresponding to the DMS is highlighted in purple.

The main purpose of this study was to perform a thorough evaluation of the potential pathogenic effect of all *h*RPE65 missense mutations labelled as variants of uncertain significance (VUS), reported in the ClinVar database, associated to residues located either within the dimer-mediating sequence (DMS) of the protein or in its close proximity. In particular, we identified 13 different VUS associated to 12 residues included in the dimerisation region of the protein (Figure 1): N301S, K303N, T306I, S307F, N373S, D375N, D375H, V384F, T390I, E399K, T400S, W402S and V407A.

For this evaluation, we aimed at employing a thorough structure-based analysis relying on MD simulations, using the validated DMS-focussed *h*RPE65 models and µs-long simulation protocols developed in our previous study. In fact, our MD-based approach demonstrated the ability to discriminate between likely pathogenic missense variants, predicted to be potentially associated to deleterious effects on local folding and conformation stability of the DMS, and experimentally-confirmed unharmful mutations, predicted to have a negligible impact from a structural point of view^15^. In particular, the results of our previous study showed the advantage of performing MD-based analyses using DMS-focussed dimeric *h*RPE65 models, composed of mutated and wild-type (WT) monomers, over employing monomeric models of *h*RPE65 variants. Specifically, the use of the dimeric models allowed to more rapidly observe the predicted deleterious effects on local protein folding associated to the likely pathogenic variant, and to better highlight the neutral impact of the benign mutation. For this reason, we decided to employ this strategy for the analysis of the 13 selected *h*RPE65 VUS.

**Figure 2.**
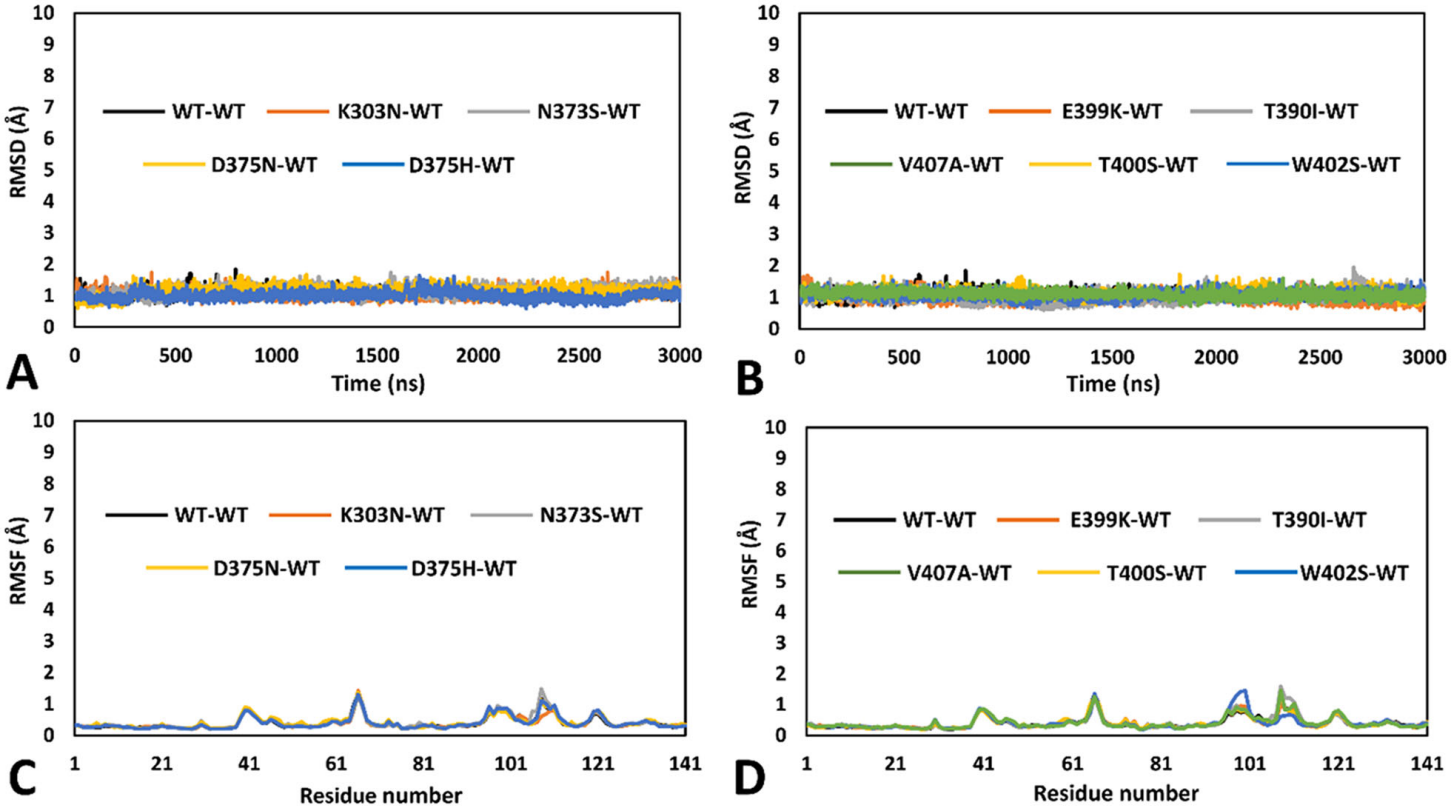
MD results obtained for the nine dimeric models of hRPE65 including K303N, N373S, D375N, D375H, E399K, T390I, V407A, T400S and W402S variants. A), B) RMSD of the DMS α carbons during 3 µs of MD simulation obtained for the mutated monomers compared to WT. C), D) RMSF of the α carbons of the whole mutant monomers compared to WT.

Initially, 13 different mutated *h*RPE65 models, one for each single variant to be analysed, were generated and combined with the previously generated WT monomeric model (see Materials and Methods for details), thus obtaining 13 different dimeric models in which one monomer presented a single specific missense mutation, whereas the other monomer corresponded to the WT structure. All different dimeric systems so obtained were then subjected to a 3 µs-long MD simulation protocol using the hydrogen mass repartitioning (HMR) scheme, as previously performed, for a total of almost 40 µs of simulation time. After the simulations, all models were initially subjected to a trajectory analysis including the evaluation of the of the root-mean-square deviation (RMSD) of the DMS α carbons and the root-mean-square fluctuation (RMSF) of the total α carbons of the mutant monomer. These results were then compared with those obtained from the analysis of the 3 µs-long MD simulation previously generated for the reference DMS-focussed WT-WT dimeric model. Moreover, we also performed a thorough MD-based analysis of the residue-specific pairwise interactions associated to the local protein environment interested by the mutation in each studied variant, compared to WT. Specifically, we analysed the MD trajectory previously generated for the reference WT-WT model for evaluating the pattern of H-bond interactions involving the residues associated to missense mutations in the 13 herein studied VUS; subsequently, we performed the same analysis on the MD trajectories obtained for each mutant-WT model, thus assessing the impact of each missense mutation on the native pattern of H-bond interactions during the MD simulation generated for each VUS. In this way, we aimed at identifying alterations of pairwise interactions that may suggest a potential subtle deleterious effect on local folding and stability that could not be otherwise observed during the MD simulation. Surprisingly, a first group comprising nine out of the thirteen VUS-based dimeric systems, including K303N, N373S, D375N, D375H, E399K, T390I, V407A, T400S and W402S variants, did not show any appreciable structural deviation and significant difference with respect to the WT model in terms of both RMSD and RMSF. As shown in Figure 2, the results of the MD trajectories obtained for these nine variants were fully comparable to those previously obtained for the WT dimer.

**Figure 3.**
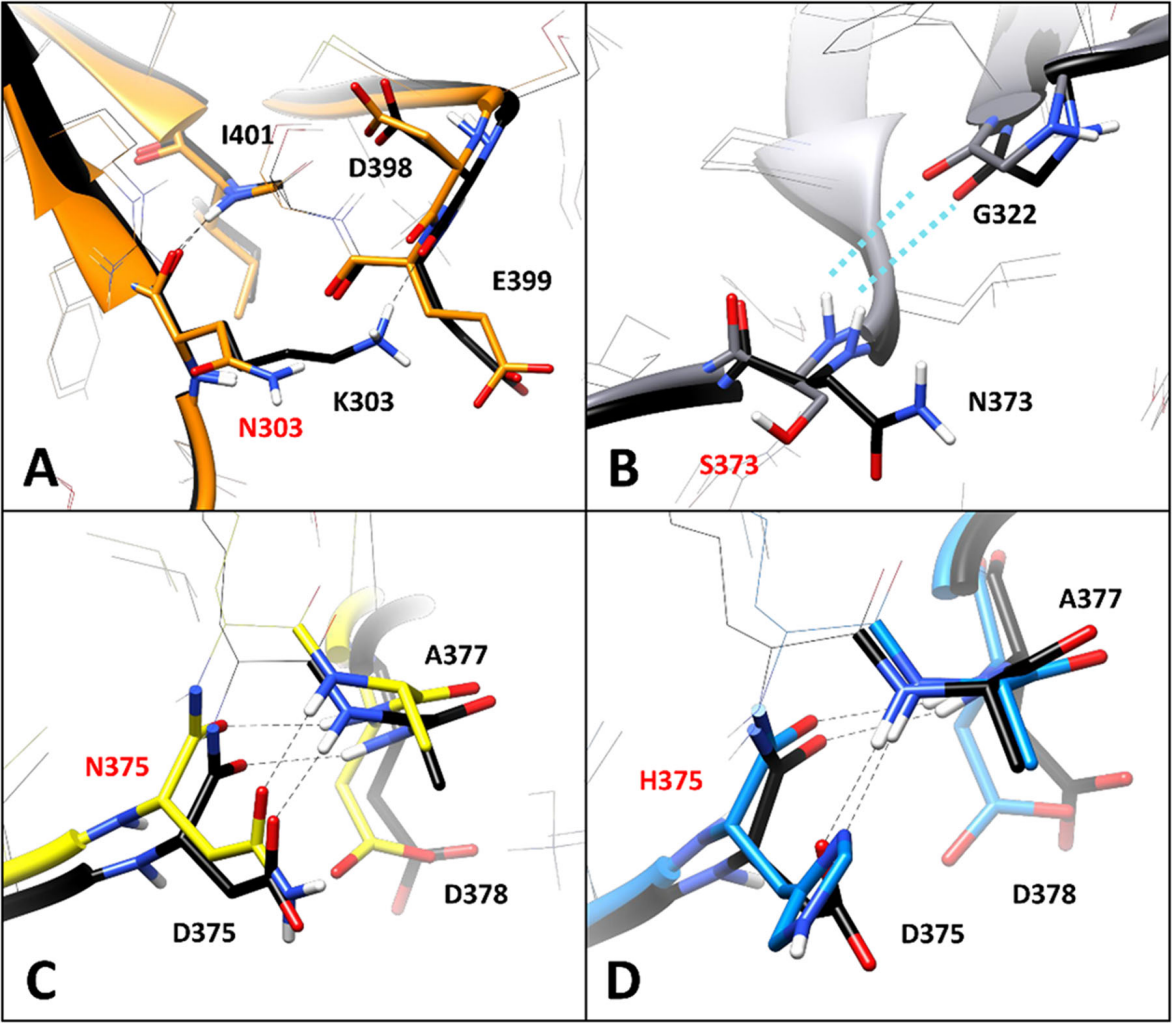
Residue-specific pairwise interactions observed for the mutated residues in the models of the analysed VUS, compared to the WT model. A) K303N (orange), B) N373S (gray), C) D375N (yellow), D) D375H (blue). All VUS structures displayed correspond to the minimised average structure obtained from the last µs of MD simulation, which are shown superimposed to the minimised average structure of the WT model (colored black in all panels). Direct H-bonds are shown as black dashed lines. Water-mediated interactions are showed as cyan dotted lines.

Nevertheless, the in-depth residue-specific pairwise interaction analysis highlighted the disruption of relevant intramolecular and intermolecular H-bonds in the some of these VUS, which suggest potentially pathogenic features. Figure 3(A) shows the minimised average structure of K303N model obtained from the last µs of MD simulation, with a focus on the mutant residue N303 and its surroundings, superimposed to the minimised average structure of the WT model. The WT residue K303 formed two main H-bond interactions with the surrounding residues: a strong H-bond established with the backbone nitrogen of I401, which was maintained for 99% of the MD, and a further interaction established by its positively charged amino group with the backbone oxygen of D398, which was observed for more than 50% of the MD. Although the interaction with I401 is conserved in the mutant K303N model, the shorter side chain of N303 does not allow to maintain the H-bond with D398. Since the interaction between K303 and D398 is a charge-assisted H-bond that could contribute to anchor the hairpin portion of the DMS beta sheet to the rest of the protein (Figure 1 and Figure 3(A)), the mutation of K303 to N303 could have potential deleterious effects on the stability of the protein. On the contrary, no alteration of specific pairwise interactions observed in the WT model was associated VUS N373S. In fact, the WT residue N373, located in the initial portion of the DMS loop (Figure 1), does not appear to form any significant interaction with the surrounding residues, except for a transient water-mediated H-bond with G322, which is however observed even in the mutated N373S model (Figure 3(B)). Consequently, the mutation of N373 to S373 should have a neutral effect in terms of pairwise interactions.

The WT residue D375 shows two different H-bonds with the backbone nitrogen of D378 and A377. The first one is formed through its backbone oxygen and maintained for about 90% of the MD, while the second one is established through its carboxylic group, and observed for about 55% of the simulation (Figure 3(C,D)). The substitution of D375 with either N375 or H375 did not show to significantly alter the pattern of interactions with A377 and D378. In fact, the interaction with the backbone nitrogen of D378 was perfectly maintained with comparable stability (85–90% of the MD), and the H-bond with the NH group of A377 could still be performed both through the carboxamide oxygen of N375 (Figure 3(C)) and the imidazole nitrogen of H375 (Figure 3(D)). Although these interactions formed by the side chains of N375 and H375 with A377 are not charge-assisted H-bonds as in the WT protein, they showed similar or even higher stability than the native interactions, being observed for about 52 and 80% of the MD, respectively. Based on this considerations, no significant elements suggesting a potential pathogenicity of the two VUS D375N and D375H were observed in this analysis.

**Figure 4.**
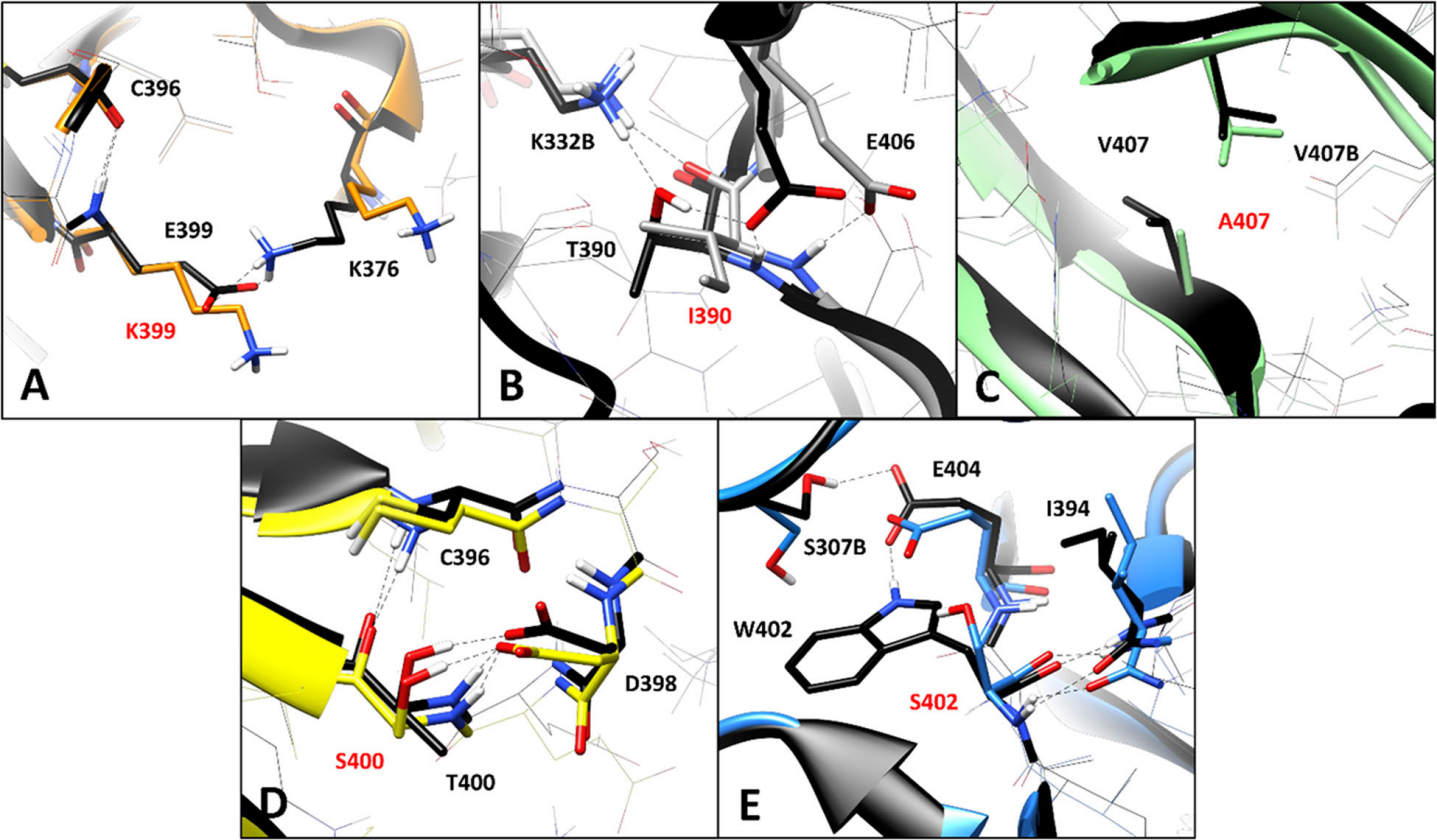
Residue-specific pairwise interactions observed for the mutated residues (labelled with red font) in the models of the analysed VUS, compared to the WT model. A) E399K (orange), B) T390I (gray), C) V407A (green), D) T400S (yellow), E) W402S (blue). All VUS structures displayed correspond to the minimised average structure obtained from the last µs of MD simulation, which are shown superimposed to the minimised average structure of the WT model (colored black in all panels). Direct H-bonds are shown as black dashed lines.

The WT residue E399, which is located in the hairpin portion of the DMS beta sheet (Figure 1), is associated to the VUS E399K. The analysis of the MD simulations of the WT system reveals two different interactions established by E399: a backbone-backbone H-bond with the carbonyl oxygen of C396 and a salt bridge observed between the carboxylic group of E399 and the positively charged amino group of K376 (Figure 4(A)), which are maintained for about 60 and 70% of the whole MD simulation, respectively. The substitution of E399 with K399 clearly disrupts the interaction with K376, although the H-bond with C396 is maintained. Considering that this mutation determines the absence of an ionic interaction which could contribute to anchor the DMS loop (in which K376 is located) to the adjacent beta sheet (Figure 1), the pairwise residue interaction analysis reveals a potential pathogenic effect associated to E399K VUS. Similarly, the substitution of T390 with an isoleucine, as occurs in VUS T390I, is supposed to have a deleterious impact on the network of intramolecular and intermolecular interactions of the *h*RPE65 dimer. T390 is located in the terminal portion of the DMS loop (Figure 1), which is exposed to the interface residues of the other monomer of the dimer. In fact, the hydroxyl group of T390 forms a stable H-bond (for more than 80% of the MD) with the positively charged amino group of K332 from monomer B (K332B, Figure 4(B)). Moreover, two additional intramolecular H-bonds are observed with the carboxylic group of E406 of the same monomer, which are formed through the backbone nitrogen and the side chain of T390; these interactions are maintained for 80 and almost 100% of the simulation, respectively. Since the alkyl side chain of I390 of the mutant *h*RPE65 cannot form any H-bond with E406 and K332B, two different stable, charge-assisted interactions are inevitably lost in VUS T390I. Although the H-bond between K332B and the OH group T390 is partially replaced by an H-bond with the carbonyl oxygen of I390, this interaction was found to be less stable, being maintained for less than 50% of the MD, compared to an 80% stability of the WT interaction. Moreover, the bulky side chain of I390 pushes away the carboxylic group of E406 destabilising the interaction with its backbone nitrogen (maintained for 75% of the MD, instead of 100% of the simulation). Therefore, the disruption of the H-bond network formed by the WT T390, observed in VUS T390I, suggests the potential pathogenicity of this variant. A completely different situation was found analysing VUS V407A and T400S. The WT residue V4007 is a located next to the last DMS residues (Figure 1) and mainly interacts only with the side chain of the same residue belonging to the other monomer of the *h*RPE65 dimer (V407B, Figure 4(C)), through hydrophobic interactions. Therefore, the mutation of V407 into an alanine, seems to only slightly reduce lipophilic contacts, without altering any key interaction for the stability of either the complex or the conformation of the mutated monomer, and thus does not appear to have potential pathogenic effect from a structure-based point of view. Similarly, VUS T400S appears to have a neutral effect in terms of intramolecular interactions. In fact, although T400 shows a network of stable H-bonds with the backbone of C396 and the carboxylic group of D398 (Figure 4(D)), maintained for at least 80% of the MD and supposed to be important for anchoring the DMS beta-sheet to the adjacent portion of the protein, the mutated residue S400 is able to efficiently maintain all interactions observed for the WT residue from both a qualitative and quantitative point of view. Therefore, this analysis suggests the non-deleterious effect of VUS T400S. On the contrary, the pairwise interaction analysis performed on VUS W402S revealed that the WT residue W402 participates in a network of H-bonds involving the adjacent E404, as well as a serine residue belonging to the other *h*RPE65 monomer (S307B, Figure 4(E)). In practice, the carboxylic group of E404 creates a bridge between the indolic NH group of W402 and the hydroxyl group of S307B, thus stabilising the dimeric complex. Moreover, the backbone amide group of W402 forms two additional H-bonds with the backbone of I394. All these interactions were maintained for more than 70% of the whole MD simulation of the WT *h*RPE65 dimer. The mutation of W402 into S402 does not affect the stability of the two backbone-backbone H-bonds with I394; however, due to the shorter side chain of S402 compared to W402, the stability of the interaction with E404 is significantly reduced (<50% of the MD) and the H-bond appears to be totally lost in the last part of the MD (Figure 4(E)). This also determines almost the complete loss of the interaction between S307B and E404, since the orientation of the carboxylic group of E404 is not stabilised anymore by the H-bond with W402, and does not maintain the proper orientation necessary to interact with S307B. Based on these considerations, the structure-based analysis suggests the potential pathogenic effect of VUS W402S.

**Figure 5.**
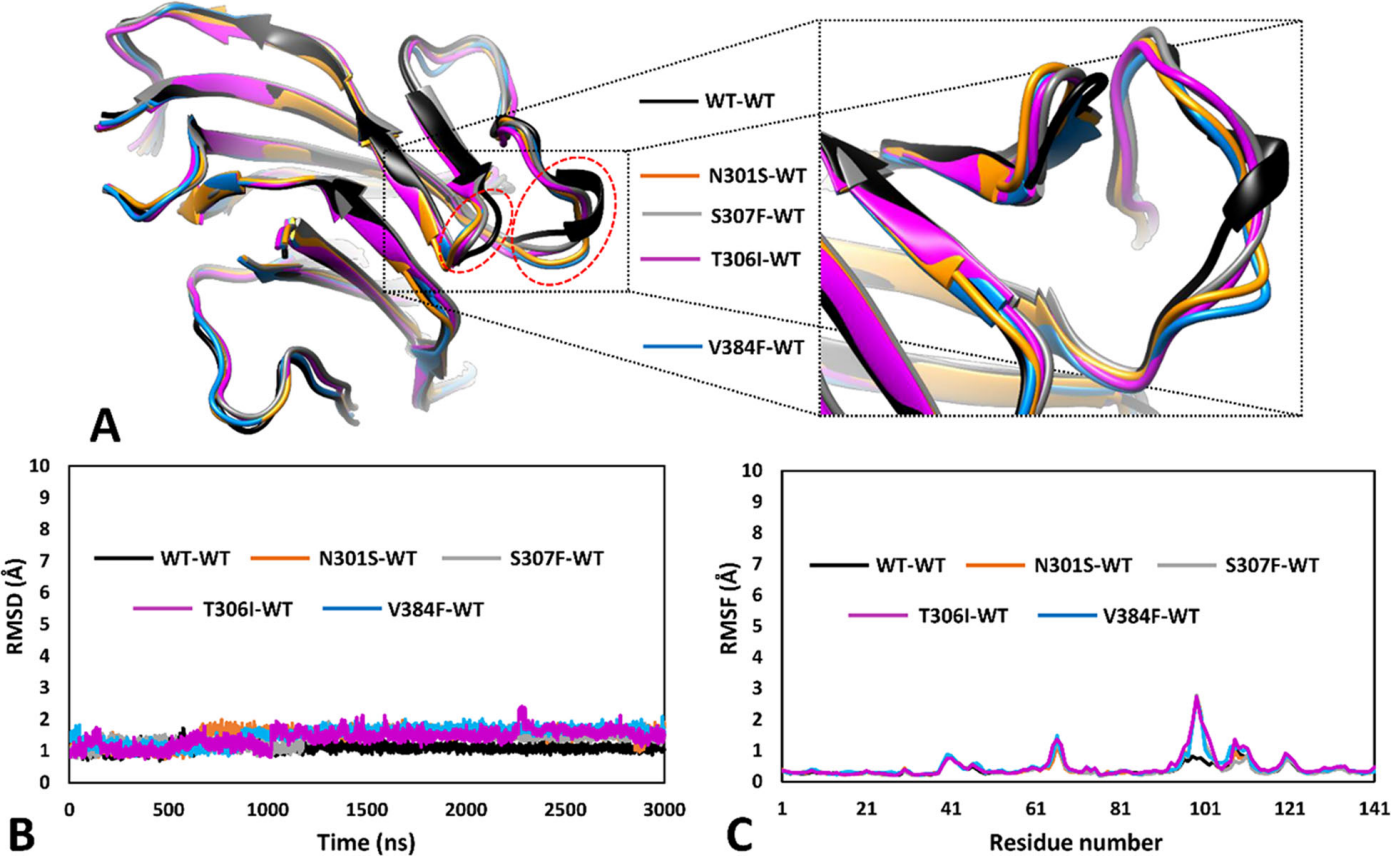
MD results obtained for the four dimeric models of *h*RPE65 including N301S, S307F, V384F and T306I variants. A) Superimposed average structures of the mutated monomers, obtained from the last µs of MD simulation, compared to the WT structure, and inset image focussed on the partially misfolded region. B) RMSD of the DMS α carbons during 3 µs of MD simulation obtained for the mutated monomers compared to WT. C) RMSF of the α carbons of the whole mutated monomers compared to WT.

The second group of VUS-based dimeric systems analysed, including the four remaining variants N301S, S307F, V384F and T306I, showed a common appreciable structural deviation with respect to the WT conformation, which was identified by the analysis of the MD results in terms of both RMSD of the DMS α carbons and RMSF of the total α carbons of the mutants (Figure 5). As shown in Figure 5(A), all four VUS presented a partial misfolding of the portion of the DMS loop (big dashed circle in Figure 5(A)) adjacent to the hairpin turn of the DMS beta-sheet (small dashed circle in Figure 5(A)), which also showed a slight movement compared to the WT structure. This was also evidenced by an appreciable increase in RMSD of the DMS α carbons of all four mutant systems, which occurred before 1.25 µs of simulation (Figure 5(B)). In fact, the average RMSD calculated for the second half of the MD of all VUS was higher than 1.5 Å, compared to the average value around 1.0 Å calculated for the WT system. The localised misfolding observed in these four VUS is better highlighted by the RMSF plots of the total α carbons of the mutant monomers, which showed a peak involving residues 98–102 of the DMS-focussed model (corresponding to residues 375–379 of *h*RPE65) of almost 3 Å, compared to the values below 1.0 Å observed for the same residues in the WT system (Figure 5(C)).

**Figure 6.**
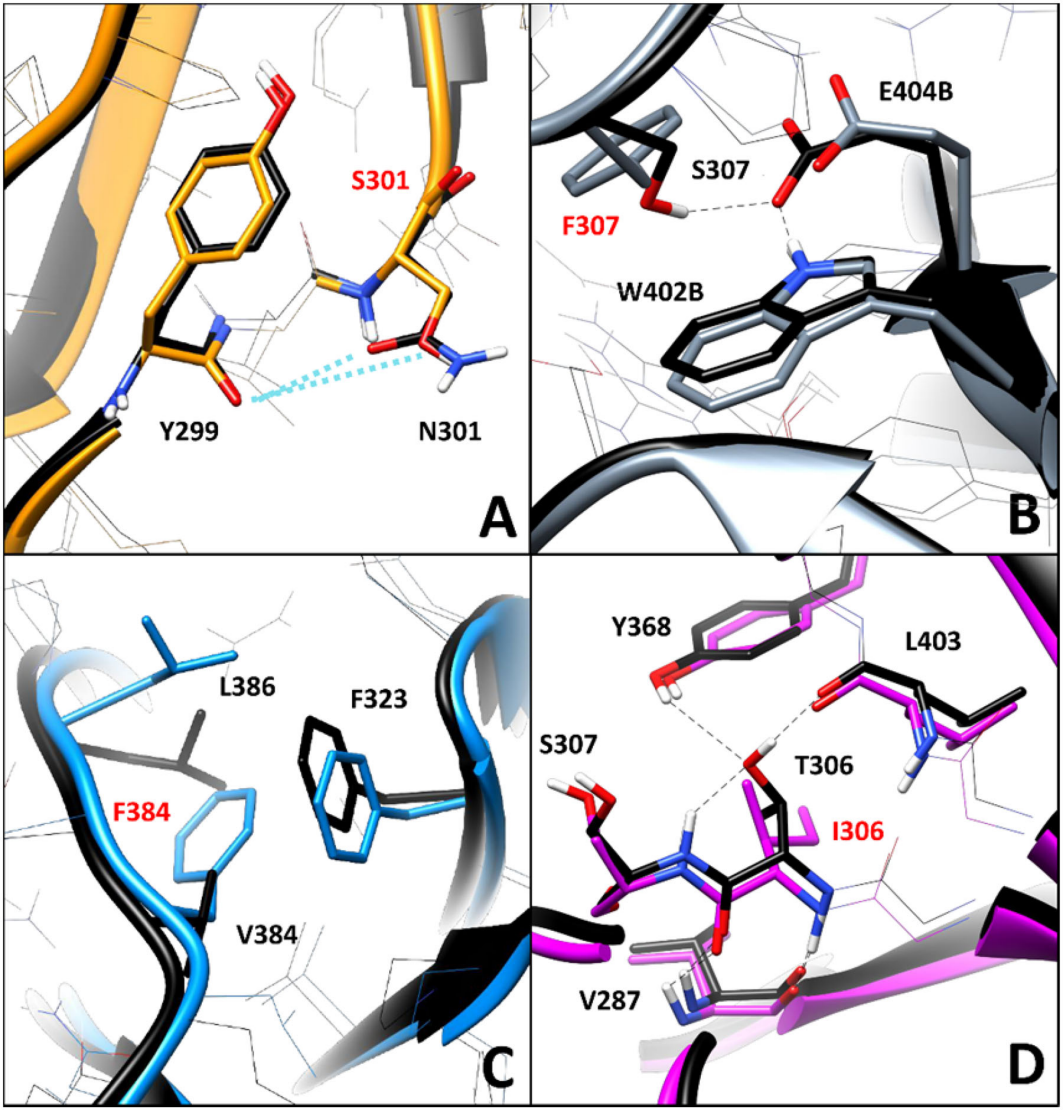
Residue-specific pairwise interactions observed for the mutated residues (labelled with red font) in the models of the analysed VUS, compared to the WT model. A) N301S (orange), B) S307F (gray), C) V384F (blue), D) T306I (magenta). All VUS structures displayed correspond to the minimised average structure obtained from the last µs of MD simulation, which are shown superimposed to the minimised average structure of the WT model (colored black in all panels). Direct H-bonds are shown as black dashed lines.

The residue-specific pairwise interaction analysis was then performed for this second group of VUS. As shown in Figure 6(A), no relevant alteration of key H-bonds was observed in variant N301S. In fact, the WT residue N301 only formed transient water-mediated interactions with Y299 that were not significantly reduced in the mutated N301S model. This suggests no deleterious effect associated to VUS N301S from a structure-based point of view. On the contrary, the mutation of S307 in F307 appears to disrupt intermolecular interactions relevant for the stability of the *h*RPE65 dimer. As discussed above, in VUS W402S the native network of H-bonds formed among W402, E404 and S307B (S307 of the other monomer) was found to be lost due to the mutation of W402 (Figure 4(E)). Similarly, as shown in Figure 6(B) (in which the same orientation of the dimeric models displayed in Figure 4(E) was maintained, for an easier comparison), the substitution of S307 with F307 not only determined the loss of the stable H-bond (maintained for 90% of the MD) with E404 of the other *h*RPE65 monomer (E404B, Figure 6(B)), but also prevented the interaction between W402B and the adjacent E404B, whose side chain was not stabilised anymore by the H-bond with S307. Therefore, VUS S307F is predicted to have a potential pathogenic feature based on this analysis. A particular scenario was observed analysing VUS V384F. The WT residue V384, which is located in the middle portion of the DMS loop (Figure 1), forms hydrophobic interactions with the lipophilic residues located in its surroundings, primarily F323 and L386 (Figure 6(C)). The substitution of V384 with a phenylalanine, as in VUS V384F, from one side appeared to alter the conformation of the side chains of the adjacent residues, especially of L386, but from the other side, the bulkier side chain of F384 seemed to form even better hydrophobic interactions with L386 and especially F323, with which a T-shaped stacking is established. For this reason, the pairwise interaction analysis does not provide clear elements to suggest a potential deleterious effect of this VUS. Finally, the WT residue T306, which is located next to S307 and close to W402 (Figure 1), appears to have an important role in the pattern of interactions contributing to the stability of the protein conformation, similarly to S307 and W402. In fact, T306 forms a network of H-bonds with the backbone oxygen of L403 (maintained for the whole MD), the hydroxyl group of Y368 and the backbone NH group of the adjacent S307 (both maintained for more than 50% of the MD). Moreover, a couple of backbone-backbone H-bonds with V287 are also observed (Figure 6(D)). The mutation of T306 in I306, as occurs in VUS T306I, clearly determines the complete loss of the H-bond network established by the OH group of T306 in the WT protein. Moreover, the presence of the bulky side chain of I306 weakens the H-bond with the backbone NH of V287 (maintained for about 60%, compared to 90% of the WT interaction). For this reason, this analysis highlights a significant pathogenic potential for VUS T306I.

### hRPE65-tailored VUS pathogenicity predictions with bioinformatics tools

In addition to the structure-based evaluation of the different VUS associated to the DMS of *h*RPE65, performed through extensive MD simulations, followed by conformational and interaction analyses, we aimed at developing an additional strategy for the prediction of the potential pathogenicity of *h*RPE65 VUS. In particular, we focussed on the development of an orthogonal approach based on the combined use of multiple bioinformatics predictive tools, which could provide complementary results to be combined with those obtained through the MD studies, for a more accurate, reliable and thorough evaluation of the potential pathogenic effect of *h*RPE65 VUS. For this purpose, we initially assessed the performance of a large set of bioinformatics tools in discriminating pathogenic from neutral missense mutations among known *h*RPE65 variants, in order to identify a subset of tools with high prediction reliability for *h*RPE65 missense mutations to be used for a *h*RPE65-tailored approach. An ensemble of almost fifty different predictive models and tools freely accessible from web servers and online platforms was selected and subjected to this analysis (see Materials and Methods for details). The ClinVar database was used as the source of known *h*RPE65 variants, from which a dataset of 30 different missense mutations classified as either *Pathogenic* or *Benign* was created and used for statistical evaluations. Unfortunately, only three missense mutations classified as *Benign* are currently reported on the ClinVar database; therefore, the dataset used was composed of 27 mutations predicted as *Pathogenic* and only 3 variants predicted as *Benign*. The dataset of known *h*RPE65 variants was used to test the predictive performance of the selected bioinformatics tools, which was evaluated in terms of Specificity, Recall, Precision and Accuracy (see Materials and Methods for details). Table S1 in Supporting Information shows the results obtained for the full set of tools tested, highlighting significant differences in their ability to identify the deleterious mutations of the dataset. In fact, the recall rates obtained ranged from 0.26, corresponding to only 7 out of 27 mutations correctly predicted as deleterious, up to the maximum value of 1.00, obtained when all 27 pathogenic mutations were correctly predicted as such. Moreover, only 4 out of all tested tools correctly identified all three benign mutations, thus obtaining a specificity value of 1.00; nevertheless, 21 tools correctly predicted two out of the three benign variants (with a specificity of 0.67), whereas the remaining tools identified either one or no neutral mutation (with a specificity of 0.33 and 0.00, respectively). Based on the obtained results, we identified 19 different tools that showed Recall and Specificity values above or equal to 0.78 and 0.67, respectively, as the most reliable ones, since they correctly identified at least 21 out of 27 pathogenic variants and at least 2 out of 3 benign mutations. As shown in Table 1, in which the statistical results obtained for the 19 selected tools are reported, all predictive methods showed Accuracy values above 0.75, thus indicating a percentage of correct predictions above 75%; moreover, all methods achieved a minimum Precision of 0.95, highlighting that almost all (at least 95% of) *h*RPE65 variants predicted as potentially pathogenic were actually classified as such in the ClinVar database.

**Table 1.** Statistical results obtained for the 19 selected bioinformatics tools.

Software	Recall	Precision	Specificity	Accuracy
Mut. Assessor	0.89	0.96	0.67	0.87
Meta-SNP	0.85	0.96	0.67	0.83
PredictSNP	0.78	0.95	0.67	0.77
PolyPhen-1	0.78	0.95	0.67	0.77
PolyPhen-2	0.85	0.96	0.67	0.83
PhD-SNP	0.85	0.96	0.67	0.83
PON-P2	0.89	0.96	0.67	0.87
PhD-SNPg	1.00	0.96	0.67	0.97
NEI Mut. Search	0.81	0.96	0.67	0.80
BayesDel_addAF	1.00	0.96	0.67	0.97
BayesDel_noAF	1.00	0.96	0.67	0.97
ClinPred	0.89	1.00	1.00	0.90
LRT	0.96	0.96	0.67	0.93
MetaRNN	1.00	1.00	1.00	1.00
MetaSVM	0.96	0.96	0.67	0.93
REVEL	1.00	0.96	0.67	0.97
CADD	1.00	0.96	0.67	0.97
Eigen-PC	1.00	0.96	0.67	0.97
Eigen	0.96	0.96	0.67	0.93

The selected set of tools, was then used for creating a consensus-based pathogenicity prediction score, named ConPath score, which combined all the different predictions obtained by the selected tools. The ConPath score, which corresponds to the sum of positive pathogenic predictions obtained with the different methods, was employed for performing the consensus pathogenicity prediction of the 13 *h*RPE65 missense VUS herein studied. Table 2 shows the results obtained for the first set of VUS analysed, i.e., K303N, N373S, D375N, D375H, E399K, T390I, V407A, T400S and W402S. The prediction generated by each of the 19 combined methods (P = pathogenic; N = Neutral), together with the ConPath score, is reported. A ConPath score ≤ 9 corresponds to a prediction of a potentially neutral effect, whereas a ConPath score ≥ 10 corresponds to a prediction of a potentially pathogenic effect. The closer the ConPath score to 19 (the maximum value), the higher the probability of pathogenic effect; the closer the ConPath score to 0 (the minimum value), the higher the probability of neutral effect. Five out of the nine VUS belonging to the first group of variants, which showed no relevant structural deviations from the WT conformation (Figure 2), were predicted as potentially neutral. In particular, N373S and D375N, which also showed no significantly deleterious effect in terms of native pairwise interactions (Figure 3), presented a ConPath score of 0 and 3, respectively, thus suggesting a high confidence in the prediction of non-pathogenicity. Similarly, D375H and T400S, which maintained the native H-bond patterns (Figure 3 and Figure 4), both obtained a ConPath score of 6. On the contrary, VUS E399K, which showed a deleterious impact on the native pattern of H-bond interactions (Figure 4(A)), was predicted as potentially neutral by the consensus bioinformatics analysis, although with the borderline ConPath score of 9, whereas VUS V407A obtained a ConPath score of 14, indicating a confident prediction of potential pathogenicity, despite showing no relevant effects in terms of intermolecular and/or intramolecular interactions (Figure 4(C)). Finally, variants K303N, W402S and T390I, which all showed to disrupt one or more charged-assisted H-bonds observed in the WT system (Figure 3 and Figure 4), were predicted as potentially pathogenic, with ConPath scores of 13, 15 and 17, respectively.

**Table 2.** Consensus bioinformatics predictions obtained for the first set of analysed *h*RPE65 VUS.

Method	K303N	N373S	D375N	D375H	E399K	T390I	V407A	T400S	W402S
Mut. Assesor	P	N	N	N	N	P	P	N	P
PhD-SNP	P	N	N	P	P	N	N	N	P
Meta-SNP	N	N	N	N	N	N	N	N	P
PredictSNP	N	N	N	N	N	P	N	N	N
PolyPhen-1	N	N	N	P	N	P	N	N	N
PolyPhen-2	P	N	N	N	N	P	N	N	N
PON-P2	P	N	N	N	N	P	P	N	N
PhD-SNPg	N	N	P	P	P	P	P	P	P
NEI Mut. Search	N	N	N	N	N	P	P	N	P
BayesDel_addAF	P	N	N	N	N	P	P	P	P
BayesDel_noAF	P	N	N	N	P	P	P	P	P
ClinPred	P	N	P	P	N	P	P	P	P
LRT	P	N	N	P	P	P	P	P	P
MetaRNN	P	N	N	N	N	P	P	N	P
MetaSVM	P	N	N	N	P	P	P	N	P
REVEL	P	N	N	N	P	P	P	P	P
CADD	P	N	P	P	P	P	P	N	P
Eigen-PC	N	N	N	N	P	P	P	N	P
Eigen	P	N	N	N	P	P	P	N	P
**ConPath Score**	**13**	**0**	**3**	**6**	**9**	**17**	**14**	**6**	**15**

Among the second group of *h*RPE65 missense variants analysed, including N301S, T306I, S307F and V384F, which all showed appreciable local structural deviations with respect to the WT conformation (Figure 5), only N301S was predicted as potentially neutral (Table 3). Precisely, a ConPath score of 1 was obtained for this variant, which highlighted a very high confidence associated to this prediction, in agreement with the neutral impact of the mutation on the native pattern of interactions observed for the WT residue (Figure 6A). On the contrary, two high-confidence predictions of potential pathogenicity were obtained for variants S307F and T306I, which showed a ConPath score of 17 and 18 respectively, consistently with the disruption of key intermolecular and intramolecular H-bond networks, respectively, as highlighted by the pairwise interaction analysis (Figure 6). Finally, VUS V384F, for which no clear element suggesting a potential deleterious effect in terms of pairwise interactions was detected (Figure 6(C)), was also predicted as potentially pathogenic by the consensus bioinformatics approach, with a ConPath score of 13.

**Table 3.** Consensus bioinformatics predictions obtained for the second set of analysed *h*RPE65 VUS.

Method	N301S	S307F	V384F	T306I
Mut. Assessor	N	P	P	P
PhD-SNP	N	N	N	P
Meta-SNP	N	P	P	N
PredictSNP	N	P	N	P
PolyPhen-1	N	P	P	P
PolyPhen-2	N	P	P	P
PON-P2	N	P	N	P
PhD-SNPg	N	P	P	P
NEI Mut. Search	N	N	P	P
BayesDel_addAF	N	P	P	P
BayesDel_noAF	N	P	P	P
ClinPred	N	P	P	P
LRT	P	P	P	P
MetaRNN	N	P	P	P
MetaSVM	N	P	P	P
REVEL	N	P	P	P
CADD	N	P	N	P
Eigen-PC	N	P	N	P
Eigen	N	P	N	P
**ConPath Score**	**1**	**17**	**13**	**18**

### Comprehensive pathogenicity evaluation of DMS-focussed hRPE65 missense VUS

The results of the MD simulations and the subsequent structure-based analyses were combined with the predictions obtained employing the consensus bioinformatics approach, in order to perform a comprehensive and multi-level evaluation of the potential pathogenic effect of the 13 *h*RPE65 missense VUS herein studied. In particular, we aimed at providing a classification of these VUS related to the probability of their pathogenic effect based on the different potentially pathogenic features highlighted by the whole study. In light of the results of the structure-based evaluations performed on the 13 different VUS-WT dimeric models, two different alerts of potential pathogenic features were identified in the studied VUS: the presence of local structural deviations from the WT conformation and the disruption of native residue-specific pairwise interactions relevant for the stability of the protein monomers and/or dimers. Obviously, a prediction of pathogenicity obtained by applying the consensus bioinformatics approach, corresponding to a ConPath score ≥ 10, was taken into account as a further alert of potential pathogenic effect of the analysed variants. Based on these considerations, the 13 *h*RPE65 VUS herein analysed were grouped into four different classes: a) VUS with high pathogenic potential (HPPV), b) VUS with moderate/high pathogenic potential (MHPPV), c) VUS with moderate/low pathogenic potential (MLPPV), d) VUS with low pathogenic potential (LPPV). In particular, each variant was considered as a HPPV, MHPPV, MLPPV and LPPV based on the presence of 3, 2, 1 and 0 pathogenic alerts, respectively, among those identified; i.e., structural alert, pairwise interaction alert and ConPath alert (ConPath score ≥ 10). The results of this classification are shown in Table 4, in which the different pathogenicity alerts observed in the studied variants are reported.

**Table 4.** Classification of DMS-focussed *h*RPE65 missense VUS based on the overall pathogenicity evaluation. The presence (+) or absence (−) of each pathogenicity alert is shown for each variant.

hRPE65 VUS	Structural Alert	Interaction Alert	ConPath Alert	Predicted Class
K303N	–	+	+	MHPPV
N373S	–	–	–	LPPV
D375N	–	–	–	LPPV
D375H	–	–	–	LPPV
E399K	–	+	–	MLPPV
T390I	–	+	+	MHPPV
V407A	–	–	+	MLPPV
T400S	–	–	–	LPPV
W402S	–	+	+	MHPPV
N301S	+	–	–	MLPPV
S307F	+	+	+	HPPV
V384F	+	–	+	MHPPV
T306I	+	+	+	HPPV

Notably, considering that none of the nine VUS belonging to the first group of analysed variants (i.e., K303N, N373S, D375N, D375H, E399K, T390I, V407A, T400S and W402S) presented a structural deviation alert, no variant belonging to this group was classified as HPPV, since no VUS was associated to all three different pathogenic alerts. In particular, the four variants N373S, D375N, D375H and T400S, which showed no type of pathogenicity alert, were classified ad LPPV; these VUS are thus suggested as potentially neutral *h*RPE65 missense variants with high confidence by the whole computational analysis. The two variants E399K and V407A, for which only a single pathogenicity alert was observed, were classified as MLPPV, whereas VUS K303N, T390I and W402S were labelled as MHPPV, showing both the interaction and the ConPath alerts. Differently, none of the four variants belonging to the second group of analysed VUS (i.e., N301S, S307F, V384F and T306I) was classified as LPPV, since all of them presented a local structural deviation from the native conformation and were thus associated to at least one pathogenic alert. VUS N301S was labelled as MLPPV, presenting no additional pathogenicity alert, whereas V384F was classified as MHPPV, since it was predicted as pathogenic by the ConPath analysis and thus showed two different alerts. Finally, the two VUS T306I and S307F presented all three different pathogenicity alerts, and were thus classified as HPPV. Therefore, based on the whole *in silico* analysis, these VUS are suggested with high confidence as potentially pathogenic *h*RPE65 missense variants.

## Conclusions

In the present work, our innovative *in silico* protocol based on µs-long MD simulation studies, calibrated and optimised for assessing the impact of missense mutations located within the DMS region of *h*RPE65 and its surroundings, was applied to perform a thorough structure-based evaluation of the potential pathogenicity of a set of 13 different *h*RPE65 VUS associated to residues located in such region of the protein. The structural analysis, assessing both local deviations from the WT conformation and alterations of the pairwise interactions associated with the mutated residue in each variant, allowed to identify structure-based alerts for some of the studied VUS that may suggest a possible pathogenic effect. In addition, we developed a novel *h*RPE65-tailored *in silico* strategy for the pathogenicity prediction of missense variants, which was obtained combining the prediction of 19 different bioinformatics tools that showed high predictive reliability when tested using *h*RPE65 missense mutations with known pathogenic/neutral effect. The results of this novel consensus bioinformatics approach, implemented into a new pathogenicity score calibrated for *h*RPE65 missense variants, named ConPath score, were combined with the complementary results of the structure-based analysis for providing a comprehensive assessment of the potential pathogenicity of the different *h*RPE65 VUS herein studied. This multi-level analysis allowed to classify the 13 *h*RPE65 VUS into four different groups based on their different probability of potential pathogenic effect. Interestingly, 6 out of 13 analysed VUS were predicted as either potentially neutral or pathogenic with high confidence, based on the whole analysis, presenting either none or all of the different pathogenicity alerts, respectively, that were identified in the study. These results provide key information that may support a possible reclassification of at least this subset of the studied *h*RPE65 VUS in relation to pathological phenotypes such as RP, LCA and SECORD^27^, helping clinicians assess the eligibility for gene therapy of patients presenting such *h*RPE65 variants. Moreover, the ConPath approach may be virtually extended to all other *h*RPE65 VUS and, in general, all possible *h*RPE65 missense mutations, thus allowing a preliminary but still highly reliable evaluation of the potential pathogenicity of *h*RPE65 missense mutations. Similarly, the structure-based approach could be applied to other domains of *h*RPE65, for implementing a whole multi-level evaluation calibrated for a different set of *h*RPE65 VUS, associated to missense mutations localised in other structural portions of the protein. Finally, our strategy may be applied for developing target-tailored structure-based protocols and consensus bioinformatics predictions optimised for evaluating the impact of missense mutations on different protein targets, whose aberrant function and/or expression is implied in the development of inherited diseases.

## Supplementary Material

Supplemental Material
