# Supplementary material for: An in silico toolbox for the prediction of the potential pathogenic effects of missense mutations in the dimeric region of hRPE65
Source: J Enzyme Inhib Med Chem. 2023 Jan 11;38(1):2162047. doi: 10.1080/14756366.2022.2162047 (PMC9848331; doi:10.1080/14756366.2022.2162047)
Supplement: Supplemental Material [file IENZ_A_2162047_SM0363.pdf]

Supporting Information

An in silico toolbox for the prediction of the potential pathogenic effects of missense mutations in the dimeric region of *h*RPE65

Giulio Poli,[1] Gian Carlo Demontis,[1] Andrea Sodi,[2] Alessandro Saba,[3] Stanislao Rizzo,[4,5,6] Marco Macchia,[1] Tiziano Tuccinardi[1,*]

[1]Department of Pharmacy, University of Pisa, Pisa, Italy, [2]Department of Neurosciences, Psychology, Drug Research and Child Health Eye Clinic, University of Florence, AOU Careggi, Florence, Italy, [3]Department of Surgical Pathology, Molecular Medicine and of the Critical Area, University of Pisa, Pisa, Italy, [4]Ophthalmology Unit, Fondazione Policlinico Universitario A. Gemelli IRCCS, Rome, Italy, [5]Catholic University Sacro Cuore, Rome, Italy, [6]Consiglio Nazionale delle Ricerche, Istituto di Neuroscienze, Pisa, Italy.

Address for correspondence: Tiziano Tuccinardi, Department of Pharmacy, University of Pisa, Via Bonanno 6, 56126 Pisa, Italy. E-mail: tiziano.tuccinardi@unipi.it

Table of Contents

Table S1. Statistical results obtained for the whole set of bioinformatics tools tested. Page S2

Information Classification: General

Table S1. Statistical results obtained for the whole set of bioinformatics tools tested.

Software	TP	FP	TN	FN	Recall	Precision	Specificity	Accuracy
PANTHER	27	2	1	0	1.00	0.93	0.33	0.93
FATHMM	27	3	0	0	1.00	0.90	0.00	0.90
PROVEAN	20	1	2	7	0.74	0.95	0.67	0.73
Mut. Assessor	24	1	2	3	0.89	0.96	0.67	0.87
SIFT	21	2	1	6	0.78	0.91	0.33	0.73
SNAP2	18	1	2	9	0.67	0.95	0.67	0.67
Meta-SNP	23	1	2	4	0.85	0.96	0.67	0.83
PredictSNP	21	1	2	6	0.78	0.95	0.67	0.77
MAPP	20	1	2	7	0.74	0.95	0.67	0.73
PolyPhen-1	21	1	2	6	0.78	0.95	0.67	0.77
PolyPhen-2	23	1	2	4	0.85	0.96	0.67	0.83
SNAP	20	1	2	7	0.74	0.95	0.67	0.73
PhD-SNP	23	1	2	4	0.85	0.96	0.67	0.83
PON-P2	24	1	2	3	0.89	0.96	0.67	0.87
LIST-S2	25	2	1	2	0.93	0.93	0.33	0.87
PMut	24	2	1	3	0.89	0.92	0.33	0.83
SAAFEC-SEQ	27	3	0	0	1.00	0.90	0.00	0.90
PhD-SNPg	27	1	2	0	1.00	0.96	0.67	0.97
INPS-sequence	25	2	1	2	0.93	0.93	0.33	0.87
SuSPect	11	0	3	16	0.41	1.00	1.00	0.47
MutPred2	27	2	1	0	1.00	0.93	0.33	0.93
SNPs3D	22	2	1	5	0.81	0.92	0.33	0.77
Mupro	25	3	0	2	0.93	0.89	0.00	0.83
NEI Mut. Search	22	1	2	5	0.81	0.96	0.67	0.80
PROST	25	2	1	2	0.93	0.93	0.33	0.87
BayesDel_addAF	27	1	2	0	1.00	0.96	0.67	0.97
BayesDel_noAF	27	1	2	0	1.00	0.96	0.67	0.97
ClinPred	24	0	3	3	0.89	1.00	1.00	0.90
DANN	27	2	1	0	1.00	0.93	0.33	0.93
DEOGEN2	27	3	0	0	1.00	0.90	0.00	0.90
LRT	26	1	2	1	0.96	0.96	0.67	0.93
MetaLR	27	3	0	0	1.00	0.90	0.00	0.90
MetaRNN	27	0	3	0	1.00	1.00	1.00	1.00
MetaSVM	26	1	2	1	0.96	0.96	0.67	0.93
MutationTaster	27	2	1	0	1.00	0.93	0.33	0.93
PrimateAI	7	0	3	20	0.26	1.00	1.00	0.33
REVEL	27	1	2	0	1.00	0.96	0.67	0.97
VEST4	27	2	1	0	1.00	0.93	0.33	0.93
Fathmm-MKL	27	3	0	0	1.00	0.90	0.00	0.90
Fathmm-XF	27	2	1	0	1.00	0.93	0.33	0.93
CADD	27	1	2	0	1.00	0.96	0.67	0.97
CADD hg19	27	3	0	5	1.00	0.90	0.00	0.90
Eigen-PC	27	1	2	0	1.00	0.96	0.67	0.97
Eigen	26	1	2	1	0.96	0.96	0.67	0.93
MVP	27	3	0	0	1.00	0.90	0.00	0.90
Int. FitCons	27	3	0	0	1.00	0.90	0.00	0.90

Information Classification: General
